# Irradiation Promotes an M2 Macrophage Phenotype in Tumor Hypoxia

**DOI:** 10.3389/fonc.2012.00089

**Published:** 2012-08-06

**Authors:** Chi-Shiun Chiang, Sheng Yung Fu, Shu-Chi Wang, Ching-Fang Yu, Fang-Hsin Chen, Chi-Min Lin, Ji-Hong Hong

**Affiliations:** ^1^Department of Biomedical Engineering and Environmental Sciences, National Tsing Hua UniversityHsinchu, Taiwan; ^2^Department of Radiation Oncology, Chang Gung Memorial HospitalTao-Yuan, Taiwan

**Keywords:** radiation, tumor-associated macrophages, tumor microenvironment

## Abstract

Macrophages display different phenotypes with distinct functions and can rapidly respond to environmental changes. Previous studies on TRAMP-C1 tumor model have shown that irradiation has a strong impact on tumor microenvironments. The major changes include the decrease of microvascular density, the increase of avascular hypoxia, and the aggregation of tumor-associated macrophages in avascular hypoxic regions. Similar changes were observed no matter the irradiation was given to tissue bed before tumor implantation (pre-IR tumors), or to established tumors (IR tumors). Recent results on three murine tumors, TRAMP-C1 prostate adenocarcinoma, ALTS1C1 astrocytoma, and GL261 glioma, further demonstrate that different phenotypes of inflammatory cells are spatially distributed into different microenvironments in both IR and pre-IR tumors. Regions with avascular hypoxia and central necrosis have CD11b^high^/Gr-1+ neutrophils in the center of the necrotic area. Next to them are CD11b^low^/F4/80+ macrophages that sit at the junctions between central necrotic and surrounding hypoxic regions. The majority of cells in the hypoxic regions are CD11b^low^/CD68+ macrophages. These inflammatory cell populations express different levels of Arg I. This distribution pattern, except for neutrophils, is not observed in tumors receiving chemotherapy or an anti-angiogenesis agent which also lead to avascular hypoxia. This unique distribution pattern of inflammatory cells in IR tumor sites is interfered with by targeting the expression of a chemokine protein, SDF-1α, by tumor cells, and this also increases radiation-induced tumor growth delay. This indicates that irradiated-hypoxia tissues have distinct tumor microenvironments that favor the development of M2 macrophages and that is affected by the levels of tumor-secreted SDF-1α.

## Introduction

A major obstacle in cancer radiation therapy (RT) or chemotherapy is the presence of hypoxic tumors, and this could be an even more serious issue in recurrent tumors in which the hypoxia can shift from transient to chronic hypoxia (Chen et al., 2011). The recurrent tumors are not only less responsive to salvage RT or chemotherapy, but also have a higher risk of metastasis (Vicini et al., 2003). Although the effects of hypo-perfusion and low oxygen contents on tumor cells are often blamed for poor treatment response, distinct tumor microenvironments within hypoxic regions such as where there are more acidic or contain high numbers or distinct populations of macrophages, also play significant roles in tumor resistance to therapy (Jiang et al., 2010; Zhang et al., 2010; Denardo et al., 2011). Several new treatment protocols to target the tumor microenvironments have been suggested, such as pH responsive drug delivery (Chiu et al., 1999; Benoit et al., 2010) and macrophage-targeted (Ahn et al., 2010; Jiang et al., 2010) or -assisted (Alizadeh et al., 2010; Muthana et al., 2011) cancer therapy. However, the improvement of cancer therapy by these approaches has still to be realized. One critical issue in targeting the tumor microenvironment is that its changes during or after the therapy. This continuous and dynamic process is crucial for the right timing of intervention. We have previously shown that there are temporal and spatial changes in the subcomponents within tumor microenvironments following single or fractionated radiation (Chen et al., 2009). Better understanding of the dynamic features of hypoxic microenvironments following RT may provide new strategies to improve the efficacy of cancer treatment.

One remarkable feature in hypoxic tumor microenvironments is the large amount of infiltrated macrophages, so-called tumor-associated macrophages (TAMs). TAMs represent the largest population of infiltrating inflammatory cells in malignant tumors. They were originally thought of as one host defense mechanism against the developing cancer. However, evidence has accumulated indicating that TAMs may assist tumors to survive hazardous environments in various ways (Nishie et al., 1999; Bingle et al., 2002; Murdoch and Lewis, 2005; Lewis and Pollard, 2006; Li et al., 2007; Ahn and Brown, 2008; Qian and Pollard, 2010; Chen et al., 2011) and even promote tumor resistance to chemotherapy (Zhang et al., 2010; Denardo et al., 2011). Two distinct TAM phenotypes, M1 or M2, have been described with the abilities to inhibit or promote tumor growth, respectively. The M1 phenotype is proinflammatory and has high levels of iNOS production; the M2 phenotype (Mantovani et al., 2002) is anti-inflammatory, pro-angiogenic (Dirkx et al., 2006; Lin et al., 2006), metastasis-promoting (Leek et al., 1996; Hanada et al., 2000), and has high levels of Arg I production. However, TAMs may change their functions under different microenvironments (Stout et al., 2005; Chiang et al., 2008; Redente et al., 2010). It has been hypothesized that initial TAMs are predisposed to have M1 function, but are gradually changed to M2 function as tumor grow (Weigert and Brune, 2008). This is associated with factors, such as IL-4, IL-10, TGF-β, PGE2, and chemokines, released by tumor cells in response to the changes in the microenvironments, in particular the development of hypoxia (Mantovani et al., 1986; Lewis et al., 1999). Furthermore, TAMs within different subcomponents of the same tumor may also have different functions. Ohno et al. (2003) had shown that TAMs in different niches of gastric carcinoma have different influences on patients’ survival, stressing the heterogeneity of TAMs and the effects of tumor microenvironment on TAM function. It has been proposed that M1 and M2 TAMs will segregate to different areas of the tumor, with M2 TAMs migrating to and aggregating in avascular or hypoxia regions (Lewis and Murdoch, 2005; Murdoch and Lewis, 2005). This may explain the variation of M1/M2 ratio and inconsistency in the correlation of the number of TAMs with the prognosis in different types of tumor (Bingle et al., 2002). For example, a positive correlation has been reported in breast and prostate cancer, but a negative correlation in colon caner. Contrary conclusions from the same type of tumor had also been reported in brain tumors when different types of surface markers were used (Bingle et al., 2002). These findings indicate that host or tumor factors may be critical for assessment of the effects of tumor microenvironments on TAM function, in particular for tumor re-growth after RT because our previous studies showed TAMs are actively involved in the remodeling of post-radiation microenvironments (Chen et al., 2011). However, systematic studies on this area are lacking.

The potential of RT to alter TAM phenotype and function has rarely been studied. In our previous study, TAMs isolated from irradiated tumors expressed higher Arg I, COX-2, and iNOS levels than those from un-irradiated tumors and were more effective at promoting tumor growth (Tsai et al., 2007), indicating more M2 TAMs in irradiated-TRAMP-C1 tumors. In this study, we used three different murine tumors, TRAMP-C1 prostate adenocarcinoma, ALTS1C1 astrocytoma, and GL261 glioma, to explore how irradiation affects the relationship between hypoxia and TAMs and whether those changes in irradiated tumor microenvironments are affected by tumor or host factors.

## Materials and Methods

### Tumor model and tumor irradiation

All experiments were performed using 7- to 8-week-old male C57BL/6J mice obtained from National Laboratory Animal Center, Taiwan. The TRAMP-C1 prostate cancer cell line was derived from transgenic mice with adenocarcinoma of the mouse prostate (Foster et al., 1997) and was purchased from the ATCC (CRL-2730). ALTS1C1 was derived from primary astrocytes transformed by SV40 large T antigen and serial *in vivo* passage (Wang et al., 2012) and is deposited in Bioresource Collection and Research Center (BCRC-60582), Taiwan. GL261 was a generous gift from Prof. Newcomb, E. W., Departments of Pathology, New York University School of Medicine (Newcomb et al., 2010). For intramuscular model, tumors were generated by intramuscular inoculation of 3 × 10^6^ viable cells into the thigh. Mice with tumors of 4 mm in diameter were selected and randomly allocated to groups for experimentation (tumor diameter was defined by (*a* + *b*)/2, where *a* and *b* are the width of two dimensions of mouse thigh) that contained at least five mice per time point. To implant ALTS1C1 or GL261 cells into the brain, 2 μl containing 1 × 10^5^ cells were inoculated intracranially (i.c.) into 6- to 8-week-old C57BL/6 mice as described (Wang et al., 2012). Prior to sacrifice, the animals were anesthetized and then perfused transcardially with PBS followed by 4% paraformaldehyde. The maximum tumor cross sectional area was used to compare the tumor growth for i.c. tumor model and defined by [(*a* + *b*)/2]^2^ × π, where *a* and *b* are the width of two dimensions of maximum cross section.

The irradiation protocol was as previously described (Tsai et al., 2007). Tumors were irradiated with either a single dose of 25 Gy to the intramuscular tumor or 8 Gy to intracranial tumors. The tumors were removed at indicated times following irradiation. During the experiments, all mice were cared for in accordance with the approved guide by the Institutional Animal Care and Use Committee (IACUC), National Tsing Hua University, Taiwan (approved number: IACUC:09705).

### cDNA microarray

Total RNA was isolated by PureLink RNA purification system (Invitrogen) according to the manufacturer’s instructions to generate cRNA targets. The samples of primary astrocytes and two cell lines, ALTS1C1, and GL261, were hybridized using Affymetrix Mouse Genome 430A 2.0 Oligonucleotide Microarrays in the Genomic Medicine Research Core Laboratory (GMRCL) of Chang Gung Memorial Hospital (Wang et al., 2004). After scanning, hybridization signals were collected and the signals that were differentially expressed twice as compared with the normal astrocyte were selected for further analysis.

### RT-PCR

Total RNA was extracted with TRIzol (Invitrogen). Two micrograms of total RNA was reverse-transcribed using Super Script III RNase H reverse transcriptase (Invitrogen, CA, USA) and random hexamer primers (Invitrogen) at 25°C for 10 min and 42°C for 1 h. Two microliters of the reverse transcription product was used as a template for PCR amplification. PCR was performed using Taq polymerase (Invitrogen) and 150 nmol/L of primers. The PCR conditions consisted of 3 min of an initial denaturation step (95°C followed by 30 cycles of denaturation (95°C, 30 s), annealing (57°C, 30 s), and extension (72°C, 30 s) followed by a final elongation step of 10 min at 72°C. Ten microliters of PCR product was analyzed on 2% agarose gels stained with ethidium bromide. Quantitation of bands was done with the Bio-Rad Fluor-S apparatus (Bio-Rad, Hercules, CA, USA) with Quantity One (version 4.2.1) software.

### Immunohistochemistry

Tumor hypoxia was studied by i.v. injection of 4 mg pimonidazole hydrochloride (Hypoxyprobe^™^-1 Kit, Hypoxyprobe, Burlington, MA, USA) in 0.1 ml solution 1 h before tumor harvest. Tissues were removed and placed in cold 4% paraformaldehyde overnight then processing and embedding in paraffin or OCT. Ten micrometers cryostat sections were fixed in methanol at −20°C for 10 min, and then rehydrated in PBS. Non-specific binding was blocked by incubating sections in 1% of bovine serum albumin (BSA) in PBS for 30 min. Tumors sections were double-stained for pimonidazole in combination with CD31 or CD68. Pimonidazole (POMO) was detected with mouse antibody (Hypoxyprobe) and goat anti-mouse IgG_γ1_ Alexa 488 (Invitrogen). For endothelial cells, rat anti-CD31 antibody (BD biosciences, San Jose, CA, USA) was used, followed by goat anti-rat Alexa 594 (Invitrogen). For macrophages, rat anti-CD68 (Serotec, Raleigh, NC, USA), anti-F4/80 (Serotec), or anti-CD206 (Biolegend) was used, followed by goat anti-rat Alexa 594 (Invitrogen). Slides were rinsed in PBS and mounted with ProLong^®^ Gold anti-fade reagent (P-36931, Invitrogen).

### Image acquisition, processing, and analysis

Immunofluorescent images from each tumor section were captured using an external digital camera (Dxm 1200C, Nikon, Tokyo, Japan) on a Nikon fluorescence microscope (Nikon Eclipse TE 2000-S) or an AxioCam MRC-5 camera on an Axiovert40 fluorescence microscope (Carl Zeiss, Göttingen, Germany) and processed using Image-pro plus 6.0 software (MediaCybernetics, Bethesda, MD, USA). Microvascular density (MVD) was determined as the number of pixels positive for CD31 divided by the total tumor area. The hypoxia fraction was defined as the area positive for pimonidazole divided by the total tumor area (necrosis excluded). The density of macrophages in the hypoxic region was defined as the fraction of pixels positive for CD68 in the pimonidazole positive tumor area divided by the fraction of total CD68 positive pixels within the selected field. The mean intensity of Arg-1 of CD68 positive TAMs was calculated as the sum of intensity of Arg-1 that are double positive for Arg-1 and CD68 divided by the Arg-1 staining color pixels in selected area. The mean intensity of CD11b positive cells in control tumor was calculated as a reference. Regions with relative CD11b intensity >125 or <75% of reference CD11b mean intensity were arbitrarily defined as CD11b^high^ or CD11b^low^ regions, respectively.

### Statistics

Statistical analyses used GraphPad Prism version 3 (GraphPad Software, San Diego, CA, USA). For all comparisons, assessment of statistical significance was by unpaired *t*-tests with significance set at *P* = 0.05.

## Results

### The association of CD68+ TAMs with hypoxia is tumor and tissue dependent

**Figure 1:**
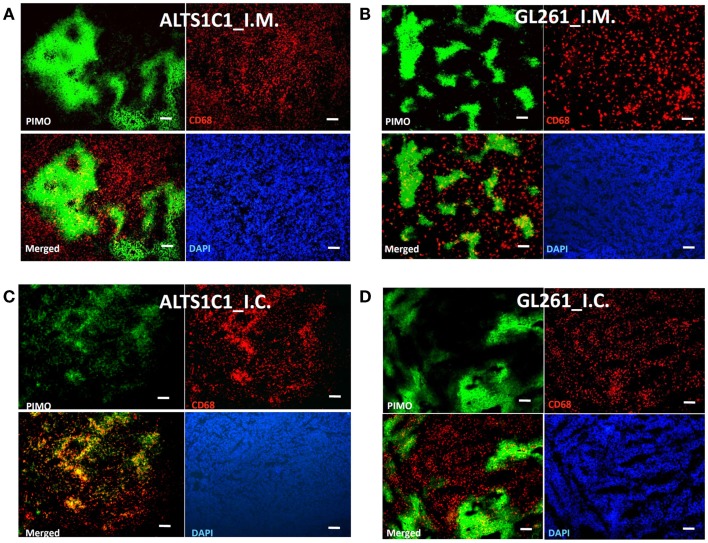
**The association of CD68+ TAMs with hypoxia is tumor and tissue dependent**. The distribution of CD68+ TAMs and PIMO+ hypoxia in ALTS1C1 astrocytoma **(A,C)** and GL261 glioma **(B,D)** grown in the thigh (i.m.) **(A,B)** or in the brain (i.c.) **(C,D)**. Green: anti-PIMO stain for hypoxic region; red: anti-CD68 antibody for TAMs. Merged images: the colocalization of hypoxia and TAMs. Scale bar = 100 μm.

Murdoch et al. (2004) have shown that CD68+ TAMs were preferably situated at hypoxic regions in a xenograft model of human cervix cancer. However, our previous study in TRAMP-C1 model has shown that CD68+ TAMs do not have any specific preference for hypoxic or non-hypoxic regions (Chen et al., 2009). To further clarify this issue, we injected murine astrocytoma, ALTS1C1, or murine glioma, GL261, murine astrocytoma and glioma, into the muscle to mimic TRAMP-C1 tumor model. The result (Figures 1A,B) clearly shows that CD68+ TAMs are randomly distributed within the tumor without any preference for the hypoxia. However, when ALTS1C1 was implanted into the brain, a degree of preference for CD68+ TAM to accumulate in hypoxic regions was found (Figure 1C), but this preference is not seen for GL261 tumors grown in the brain (Figure 1D). This result indicates that the association of CD68+ TAMs with hypoxia is tumor dependent. The differences in distribution pattern for ALTS1C1 growing in intramuscular versus intracranial sites indicates that the association of TAMs with hypoxia also depends on local environmental cues.

**Figure 2:**
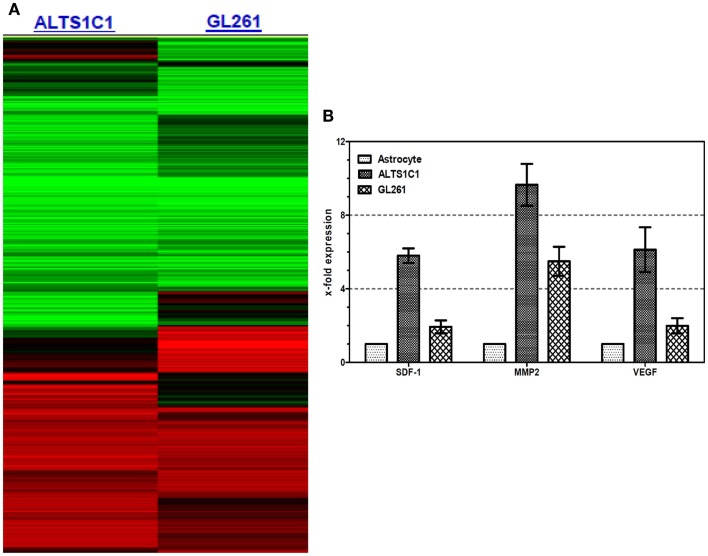
**The gene expression profiles between ALTS1C1 and GL261 cells by (A) cDNA microarray and (B) RT-PCR analysis**. The mRNA level of primary astrocyte was used as reference.

To further explore whether the association of TAMs with hypoxia is associated with factors released by tumors, a gene microarray approach was used to compare the expression profiles between ALTS1C1 and GL261 because both display different TAMs-hypoxia association pattern in the brain (Figure 2A). Following RT-PCR confirmation, the expression levels of, at least three monocyte-associated factors, SDF-1α, VEGF, and MMP-2, were identified as different between ALTS1C1 and GL261 tumor cells (Figure 2B). When the expression of SDF-1α by ALTS1C1 cells was suppressed by the transfection of lentiviral siRNA particles, the association of CD68+ TAMs with hypoxia disappeared (Wang et al., 2012). This supports the notion that factors released by tumor cells can affect the function of TAMs.

### Spatial distribution of different subsets of inflammatory cells in IR or pre-IR tumors

**Figure 3:**
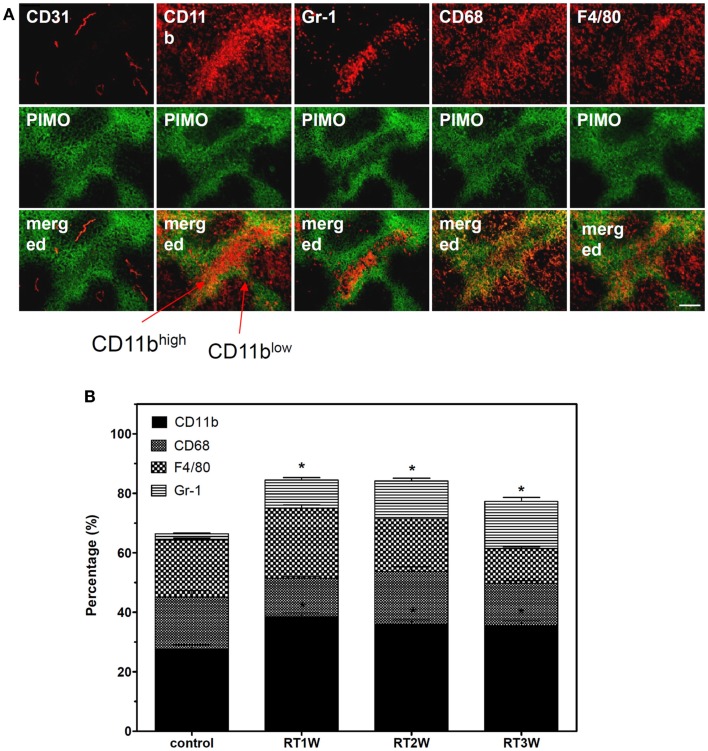
**Irradiation redistributes the localization of subtypes of inflammatory cells**. **(A)** The distribution of CD31, CD11b, Gr-1, CD68, and F4/80 staining with PIMO+ hypoxia in series 25 Gy-irradiated-TRAMP-C1 tumor sections. Green: anti-PIMO stain for hypoxic region; red: anti-CD31, anti-CD11b, anti-Gr-1, anti-CD68, or anti-F4/80 antibody. Merged images: the colocalization of hypoxia and inflammatory cells or vessels. Scale bar = 100 μm. **(B)** Percentage of CD11b, CD68, F4/80, or Gr-1 positive cells within control or 25 Gy-irradiated TRAMP-C1 tumors as assayed by flow cytometry.

An important finding from our previous study in the TRAMP-C1 tumor model was aggregation of CD68+ TAMs into chronic hypoxic regions after RT (Chen et al., 2009). These CD68+ TAMs expressed weaker CD11 staining by IHC but another subtype cells with stronger CD11 expression was found within the necrotic region (Figure 3A). These cells were further demonstrated to be Gr-1+. Based on the Gr-1 staining and intensity of CD11b expression by IHC, the CD11b+ cells can be further divided into two sub-populations, CD11b^high^/Gr-1+ neutrophils and CD11b^low^/Gr-1− TAMs. The CD11b^low^ TAMs could be further divided into CD11b^low^/CD68+ and CD11b^low^/F4/80+ TAMs. The CD11b^low^/CD68+ TAMs were highly centered in PIMO+ regions, and CD11b^low^/F4/80+ TAMs were on the edge of PIMO+ regions next to necrotic regions. In other words, this study shows that randomly distributed CD11b cells in un-irradiated control tumors re-distributed into distinct spatial location after RT. The flow cytometry assay (Figure 3B) found that the total number of CD11b cells increased after RT, but this increase was mainly the result of infiltration of Gr-1+ cells. The number of CD68+ TAMs during this period showed no significant change over a 3-week period.

**Figure 4:**
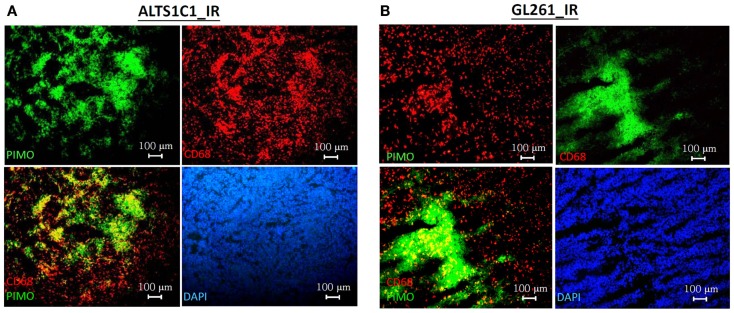
**Irradiation induces CD68+ TAMs aggregation in hypoxic regions in both ALTS1C1 and GL261 i.c. tumor models**. The distribution of CD68+ TAMs and PIMO+ hypoxia in 8 Gy-irradiated ALTS1C1 astrocytoma **(A)** and GL261 glioma **(B)** grown in the brain. Green: anti-PIMO stain for hypoxic region; red: anti-CD68 antibody for TAMs. Merged images: the colocalization of hypoxia and TAMs. Scale bar = 100 μm.

**Figure 5:**
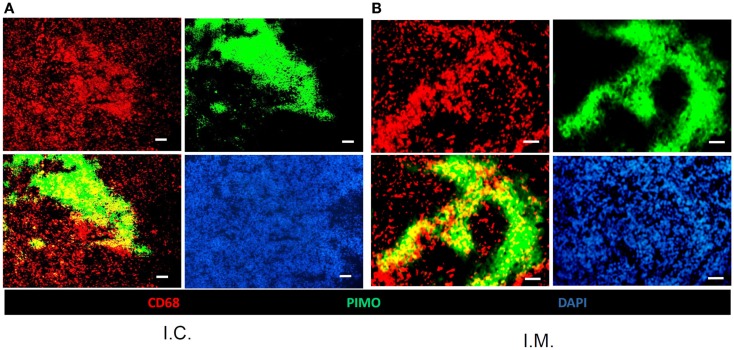
**Pre-irradiation induces CD68+ TAMs aggregation in hypoxic regions in ALTS1C1 tumors grown in both the brain (i.c.) and thigh (i.m.)**. The correlation of CD68+ TAMs with PIMO+ hypoxia in pre-irradiated ALTS1C1 astrocytoma grown in the brain (i.c.) **(A)** or in the thigh (i.m.) **(B)**. Green: anti-PIMO stain for hypoxic region; red: anti-CD68 antibody for TAMs. Merged images: the colocalization of hypoxia and TAMs. Bar: 100 μm.

The association of TAMs with hypoxic tumor before and after RT was further examined using the ALTS1C1 and GL261 brain tumor models. Figure 4 shows that RT induced the aggregation of CD68+ TAMs into PIMO+ regions in both ALTS1C1 and GL261 tumors, which was not seen in control tumors as shown in Figure 1C versus Figure 1D. This indicates that radiation-induced hypoxic regions have factors to attract or trap CD68+ TAMs. Actually, the association of CD68+ TAMs with hypoxia is not only in irradiated tumors, but also occurs in tumors growing from pre-irradiated tumor bed, so-called pre-IR tumor. This is not only seen in TRAMP-C1 tumor model (Chen et al., 2011), but also occurs in ALTS1C1 astrocytoma tumor growing in either pre-irradiated brain or muscle tissues (Figure 5).

### TAM aggregation into avascular hypoxic regions is a specific effect of irradiation

**Figure 6:**
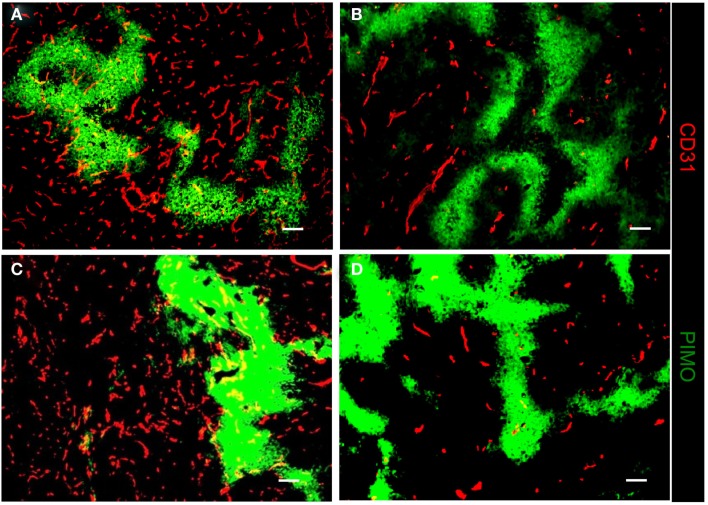
**The nature of PIMO+ hypoxic region in control and irradiated tissues is different**. The IHC staining for the distribution of hypoxia and vascular in control **(A,C)**, irradiated **(B)**, or pre-irradiated **(D)** ALTS1C1 tumors. The hypoxic regions in control tumors were vascularized. There was almost no vasculature in the hypoxic regions of irradiated **(B)** or pre-irradiated tumors **(D)**. Green: anti-PIMO stain for hypoxic region; red: anti-CD31 antibody for vessels. Merged images: the colocalization of hypoxia and vessels. Scale bar = 100 μm.

Using the pre-IR TRAMP-C1 tumor bed model, we have reported that the accumulation of TAMs is only seen in PIMO+ hypoxic areas with low MVD, but not in areas with high MVD regions (Chen et al., 2011). This challenges us to wonder whether the decrease of MVD is the prime factor for TAM aggregation. In fact, the nature of PIMO+ region in control and irradiated tissues is different (Figure 6). In control ALTS1C1 tumor, there are vessels intervening within the PIMO+ areas (Figures 6A,C) and the hypoxia is likely to be the result of vessel malfunction. On the other hand, RT destroys most vessels or alters the way they are formed within tumors (Chen et al., 2011) and the PIMO+ regions (Figures 6B,D). ALTS1C1 tumors do not contain vessels whether the tumor or the tumor bed had been irradiated. In other words, the hypoxia in irradiated tissues is likely the result of vascular insufficiency, so-called avascular chronic hypoxia. The avascular chronic hypoxia could be found in tumors receiving 25 Gy of irradiation before or after tumor implantation in both TRAMP-C1 prostate (Chen et al., 2011) and ALTS1C1 astrocytoma models (Figure 6).

**Figure 7:**
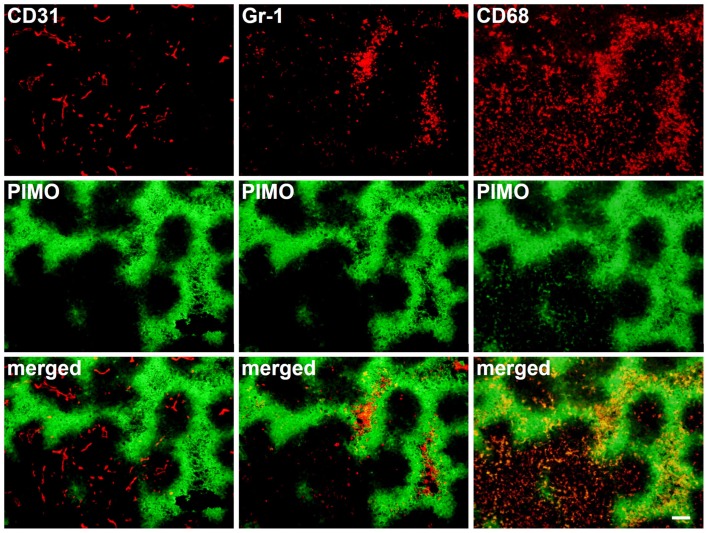
**IHC staining for CD31,Gr-1, and CD68 in series sutent-treated tumor sections**. Administration of sutent decreased vascular density and accumulated Gr-1+ cells at central necrosis in chronic hypoxia. However, CD68+ TAM does not aggregate at chronic hypoxia. Scale bar = 100 μm.

To further address the vascular issue, we used the anti-angiogenic agent, sunitinib, to treat TRAMP-C1 tumor grown in C57BL/6J mice. Anti-angiogenic agents, such as sunitinib, have been proposed as potential candidates for clinical use in recurrent tumors expressing high levels of angiogenic factors (Rauh-Hain and Penson, 2008). Our previous study in TRAMP-C1 tumors has shown that the administration of sunitinib could generate a 3-day tumor growth delay, which is less than the effect of 25 Gy of IR. Although the effects of sunitinib on growth delay were modest, dramatic changes in tumor hypoxia and MVD were found following sunitinib administration (Chen et al., 2011). Many hypoxic regions in sunitinib-treated tumors did not have vessels and were chronically hypoxic due to vascular insufficiency (Figure 7), as seen in IR tumors. The center of the avascular chronic hypoxic areas contained CD11b+ Gr-1+ cells, as was seen in IR or pre-IR tumors. However, the aggregation of CD68+ TAMs into avascular hypoxic areas that was seen in IR or pre-IR tumors did not exist in sunitinib-treated tumors (Figure 7). In fact, most CD68+ TAMs accumulated in PIMO negative regions. This demonstrates that the accumulation of CD68+ TAMs in avascular chronic hypoxia is a specific effect of RT and has less to do with the decrease in MVD or the development of avascular chronic hypoxia.

### Hypoxia-associated TAMs express higher level of Arg I

**Figure 8:**
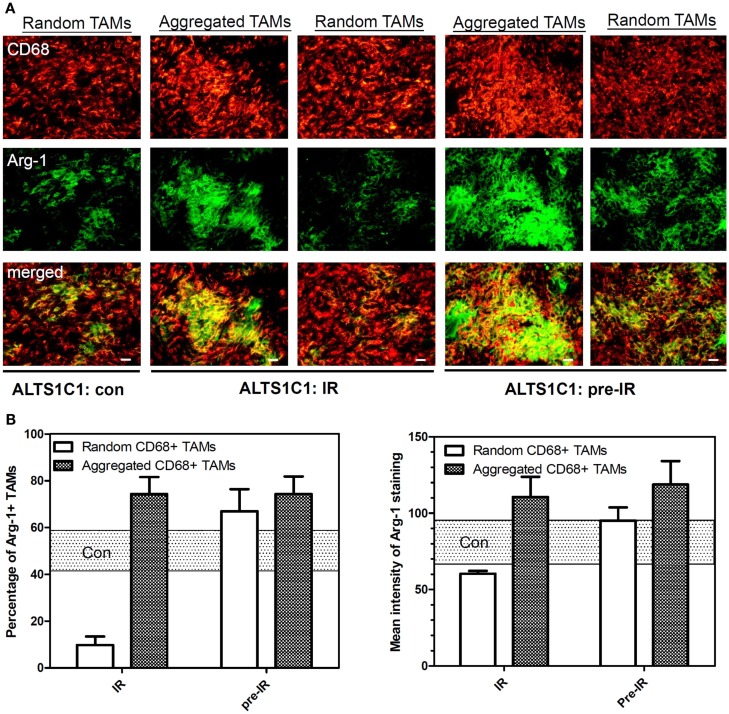
**Aggregated TAMs express higher level of Arg-1 than random TAMs**. **(A)** The IHC staining for the expression of Arg-1 by random or aggregated CD68+ TAMs in control, irradiated (IR) or pre-irradiated (pre-IR) ALTS1C1 tumor Green: anti-Arg-1 antibody; red: anti-CD68 antibody. Merged images: the colocalization of Arg-1 and TAMs. Scale bar = 100 μm. **(B)** The percentage of Arg-1+ TAMs (left graph) and the mean intensity of Arg-1 staining (right graph) by random or aggregated CD68+ TAMs in control (rectangular dot region), irradiated (IR) (white bar) or pre-irradiated (pre-IR) (dark dot bar) ALTS1C1 tumors. The rectangular dot region represents the average value ± SD in control ALTS1C1 tumor for the purpose of clarity.

Our early study using whole tumors has shown that CD11b+ TAMs isolated from irradiated tumors express higher level of Arg I and had tumor-promoting activity (Tsai et al., 2007). However, that approach cannot identify local environmental effects. In this study, the expression of Arg-1 by randomly distributed-CD68+ TAMs (i.e., at PIMO− non-hypoxic regions) versus aggregated CD68+ TAMs (i.e., at avascular PIMO+ hypoxic regions) was further verified in the ALTS1C1 intracranial model by IHC. Figure 8 shows CD68 and Arg-1 double staining in fluorescent imaging taken from control, IR, or pre-IR tumor samples with the same exposure time. It shows a higher percentage and intensity of Arg-1 staining in hypoxia-aggregated CD68+ TAMs than CD68+ TAMs that are not aggregated in hypoxic area (Figure 8B). This indicates that hypoxia-aggregated TAMs could be more polarized toward an M2 phenotype.

### SDF-1α plays a critical role in TAM aggregation and tumor re-growth after RT

**Figure 9:**
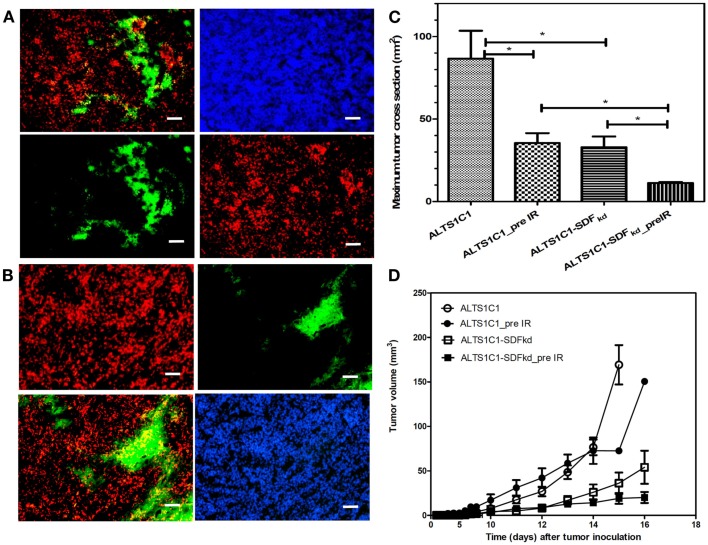
**Suppression of SDF-1 expression by ALTS1C1 tumor disrupts pre-IR-induced TAM association with hypoxia in both i.c. (A) and i.m. (B) models and prolong pre-IR-induced growth delay in both i.c. (C) and i.m. (D) models**. Green: anti-PIMO stain for hypoxic region; red: anti-CD68 antibody for TAMs. Merged images: the colocalization of hypoxia and TAMs. Scale bar = 100 μm.

SDF-1α production by ALTS1C1 is a critical factor for the accumulation of TAMs in hypoxia (Wang et al., 2012). Knock down of SDF-1(SDF^kd^) in ALTS1C1 tumors growing in a pre-irradiated tissue also inhibited CD68+ TAM aggregation whether they were grown i.c. (Figure 9A) or i.m. (Figure 9B). Tumor growth delay was also further enhanced in both i.c. (Figure 9C) and i.m. (Figure 9D) models when the expression of SDF-1α by ALTS1C1 was suppressed by siRNA. This is further indirect evidence to support the view that radiation-induced TAM aggregation in hypoxic areas stimulates tumor growth through SDF-1 production.

## Discussion

The interplay between TAMs and hypoxia is thought to be bi-directional. Hypoxia-induced HIF-1α stabilization leads to the expression of various angiogenic factors, such as VEGF, and chemotactic factors, such as SDF-1α and CSF-1, by hypoxic tumor cells. These factors further recruit peripheral macrophages to the hypoxic regions to restore blood delivery and nourish the hypoxic cells. The association of TAMs with hypoxia was originally thought to be a natural link as is indicated by several renowned publications (Leek et al., 1999; Lewis et al., 1999, 2000; Crowther et al., 2001; Burke et al., 2002; Murdoch and Lewis, 2005; Murdoch et al., 2005; Degrossoli and Giorgio, 2007; Corzo et al., 2010). However, our previous study in murine TRAMP-C1 prostate adenocarcinoma demonstrated that the CD68+ TAMs have no preference for PIMO+ hypoxia region in the control, untreated tumors (Chen et al., 2009). On the other hand, CD68+, but not F4/80+, TAMs prefer to accumulate in PIMO+ hypoxia in intracranial ALTS1C1 astrocytoma. It appears that the association of CD68+ TAMs with hypoxia is tumor dependent. This concept is further supported here by another murine brain glioma, GL261, in which CD68+ TAMs do not have a preference for PIMO+ hypoxic regions. It becomes more interesting when ALTS1C1 astrocytomas were inoculated in the muscle, where the association of TAMs with hypoxia seen in intracranial model disappeared. This indicates that the association of TAMs with hypoxia is not only tumor type dependent, but also stroma dependent. In other words, both tumor-released factors and environmental cues determine TAM function.

In this study, we used ALTS1C1 and GL262 tumors models to demonstrate that the hypoxic regions in irradiated tumors or tumors growing in pre-irradiated tissues had more CD68+ TAM accumulation than control tumors. These results are in agreement with our previous studies in TRAMP-C1 tumor model and demonstrate their reproducibility in several tumors. Most PIMO+ hypoxic regions in control tumors contain CD31+ vessels, suggesting that hypoxia resulted from abnormal vessel perfusion and these may be transiently hypoxic. On the other hand, most radiation-induced hypoxic regions did not contain CD31+ vessels, indicating that the hypoxia is caused by insufficient blood vessels and may be avascular chronic hypoxia. These radiation-induced hypoxic regions frequently develop central necrosis and are filled by Gr-1+ neutrophils. In fact, avascular chronic hypoxia could be occasionally found in larger control tumors, but no CD68+ TAM aggregation were found (Fu, S. Y. manuscript in preparation). This indicates that radiation-induced hypoxic environments have specific factors that cause CD68+ TAM aggregation. This was further supported by the use of the anti-angiogenic agent sunitinib, a tyrosine kinase inhibitor that interrupts the signaling pathways of endothelial growth factor receptor 1-3 and PDGF receptors α and β. The sunitinib-treated tumors display the decrease of MVD and the increase of avascular chronic hypoxia filled with Gr-1+ neutrophia as was seen in irradiated tumors. However, CD68+ TAMs do not accumulate in the avascular chronic hypoxic region of sunitinib-treated tumors. Instead, even more CD68+ TAMs were found in PIMO negative regions. This supports the notion that irradiated hypoxic regions have factors to cause CD68+ TAM aggregation. Since the ALTS1C1 and GL261 tumors have different CD68+ TAMs-hypoxia association patterns, microarray and RT-PCR techniques were used to isolate the genes responsible. At least three monocytes-associated factors, SDF-1α, VEGF, and MMP-2, were candidates. These factors have been separately reported to be chemoattractant for macrophages (Lewis et al., 2000; Gazitt and Akay, 2004; Kang et al., 2010) and induced by hypoxia (Burke et al., 2003; Ide et al., 2006; Williams et al., 2006; Zagzag et al., 2006; Chen et al., 2009; Wang et al., 2012). Previous studies have shown that RT can induce SDF-1α production to promote the homing of hematopoietic progenitor cells toward gliomas and enhance vessel formation (Tabatabai et al., 2006; Kioi et al., 2010). We have also found that SDF-1α in the conditioned medium produced by ALTS1C1 astrocytoma not only enhance macrophage migration toward hypoxia, but also prolong their survival in hypoxic condition (Wang et al., 2012). The current study further demonstrates that SDF-1α production by tumor cells is one of factors that are responsible for the accumulation of TAMs in radiation-induced hypoxic regions as its knock down in ALTS1C1 tumor growing in intramuscular or intracranial pre-irradiated sites prevented TAM accumulation in hypoxia. What was more interesting was that the tumor growth delay was further enhanced in SDF-1α-suppressed tumors. This implies: (1) that SDF-1α promotes tumor growth in an irradiated microenvironment or (2) that the association of TAMs with hypoxia enhances tumor growth rate. If the latter is the case, it also indicates that hypoxia-aggregated TAMs have an M2 phenotype, which is supported by Arg I staining being greater (both number and intensity) in TAMs aggregated in hypoxic than non-hypoxic regions. This further enhances the general view that TAMs in radiation-induced hypoxia are of the M2 type and have better tumor-promoting function.

In addition, this study also demonstrates that RT, no matter given before or after tumor implantation, alters tumor microenvironments so that not TAM aggregate in hypoxic regions, and Gr-1 positive neutrophils, CD68+ TAMs, and F4/80+ TAMs resegregate into different microenvironments. We believe that this is the first report to show that three subtypes of monocytic cells have their own niches in the irradiated tumor microenvironment; these cells probably play different roles based on their different locations, although we do not clearly know these roles at the present. It is reasonable to speculate that the role of Gr-1 positive neutrophils in the central necrotic regions is to clean the debris in this area. The CD68+ TAMs within avascular chronic hypoxia are likely associated with their M2 functions. The roles or functions of F4/80+ TAMs at the junction of avascular hypoxia and central necrosis need better understanding. However, it needs to caution that these changes following RT are stage dependent because the vascular damage and chronic hypoxia following RT are dynamic, which depends on radiation dose, tissues, and factors released by the tumors. For example, the maximum hypoxia-induced TAM re-segregation for TRAMP-C1 prostate tumor or ALTS1C1 astrocytoma grown in the thigh following 25 Gy of radiation was at 3 week after RT. The maximum effect for ALTS1C1 astrocytoma grown intracranially following 8 or 15 Gy of radiations was at 3 or 2 week, respectively, after RT (Wang, S. C. manuscript in preparation).

At the end, we have sorted out the relationship among tumor cells, tumor microenvironments, and tumor response to RT. We conclude that factors released from tumor cells are prime factors for the formation of specific type of tumor microenvironments such as the association of TAMs with hypoxia. Among the tumor microenvironments, MVD is the prime factor determining the nature of hypoxia and the distribution of TAMs. Following irradiation, radiation-induced tissue damage may release factors, such as SDF-1α, that dominate the effects of original tumor- or stroma-released factors. More importantly, this study shows that the aggregation of CD68+ TAMs into hypoxic regions is associated with the re-growth rate. This is further evidence to support the view that CD68+ TAMs associated with chronic hypoxia are likely M2 TAMs and a target for enhancing the efficacy of RT. Tumor-secreted SDF-1α may not be the only factor responsible for the TAM accumulation in hypoxic regions. Several studies have also shown that hypoxia-induced iNOS expression can also promote TAM migration (Weigert and Brune, 2008; Zhou et al., 2009). However, the story for iNOS may be more complex than SDF-1α because the hypoxic regions have limited supply for oxygen, the iNOS substrate (Robinson et al., 2011).

## Conflict of Interest Statement

The authors declare that the research was conducted in the absence of any commercial or financial relationships that could be construed as a potential conflict of interest.
